# Evaluating an acoustically quiet EPI sequence for use in fMRI studies of speech and auditory processing

**DOI:** 10.1016/j.neuroimage.2010.05.015

**Published:** 2010-10-01

**Authors:** Jonathan E. Peelle, Rowena J. Eason, Sebastian Schmitter, Christian Schwarzbauer, Matthew H. Davis

**Affiliations:** a MRC Cognition and Brain Sciences Unit, 15 Chaucer Road, Cambridge CB2 7EF, UK; b German Cancer Research Center, Heidelberg, Germany

**Keywords:** Sparse imaging, Quiet EPI, BOLD sensitivity, Listening effort

## Abstract

Echoplanar MRI is associated with significant acoustic noise, which can interfere with the presentation of auditory stimuli, create a more challenging listening environment, and increase discomfort felt by participants. Here we investigate a scanning sequence that significantly reduces the amplitude of acoustic noise associated with echoplanar imaging (EPI). This is accomplished using a constant phase encoding gradient and a sinusoidal readout echo train to produce a narrow-band acoustic frequency spectrum, which is adapted to the scanner's frequency response function by choosing an optimum gradient switching frequency. To evaluate the effect of these nonstandard parameters we conducted a speech experiment comparing four different EPI sequences: Quiet, Sparse, Standard, and Matched Standard (using the same readout duration as Quiet). For each sequence participants listened to sentences and signal-correlated noise (SCN), which provides an unintelligible amplitude-matched control condition. We used BOLD sensitivity maps to quantify sensitivity loss caused by the longer EPI readout duration used in the Quiet and Matched Standard EPI sequences. We found that the Quiet sequence provided more robust activation for SCN in primary auditory areas and comparable activation in frontal and temporal regions for Sentences > SCN, but less sentence-related activity in inferotemporal cortex. The increased listening effort associated with the louder Standard sequence relative to the Quiet sequence resulted in increased activation in the left temporal and inferior parietal cortices. Together, these results suggest that the Quiet sequence is suitable, and perhaps preferable, for many auditory studies. However, its applicability depends on the specific brain regions of interest.

## Introduction

During echoplanar imaging (EPI), rapid switching causes the gradient coils to oscillate, resulting in the significant acoustic noise typically associated with fMRI scanning (Price et al., 2001; Ravicz et al., 2000). This acoustic noise, which can exceed 100 dBA, presents several serious challenges for studying the processing of auditory stimuli (Moelker and Pattynama, 2003). First, and perhaps most obviously, the high sound levels generated can render experimental stimuli unintelligible. Second, even if it is possible to hear the stimuli, listeners must separate them from the noise of the scanner, adding additional perceptual and cognitive demands to the task—that is, increases in listening effort (Davis and Johnsrude, 2003). Such task demands are likely to differentially affect participants with difficulties in auditory processing due to hearing impairment or normal aging (Grimault et al., 2001; Peelle and Wingfield, 2005; Wingfield et al., 2006). Finally, the acoustic noise of the scanner itself will activate auditory cortex (Bandettini et al., 1998), which may diminish effects induced by experimental manipulations (Elliott et al., 1999; Gaab et al., 2007). Thus, standard EPI sequences are sub-optimal for auditory tasks, and may make any results difficult to interpret. In addition, the reduction of acoustic noise may also be desirable for participant comfort, particularly when dealing with children or other special populations.

One common solution to the challenge posed by acoustic scanner noise is to use a sparse imaging procedure in which the repetition time (TR) of a sequence is longer than its acquisition time (TA), clustering slice acquisition in time in order to provide a silent period between the acquisition of consecutive volumes (Edmister et al., 1999; Hall et al., 1999; Scheffler et al., 1998). Auditory stimuli can then be presented during these silent periods without disruption from echoplanar scanner noise; the delay in the hemodynamic response to a stimulus enables the BOLD signal changes associated with these stimuli to be measured by the next volume of data acquired. Because of the longer TR, for a constant amount of scanning time, fewer images are acquired in a sparse imaging paradigm than in a continuous paradigm. This approach therefore reduces the temporal resolution of the data and, due to there being fewer observations, potentially reduces the accuracy of the parameter estimates (although this may be offset by higher overall levels of signal due to the absence of spin history effects). Some modifications to sparse imaging paradigms have been developed to compensate for these shortfalls by collecting multiple volumes following a silent period (e.g., Schwarzbauer et al., 2006). Nonetheless, for a given amount of scanning time, sparse imaging approaches are fundamentally limited in the number of volumes that can be acquired relative to continuous sequences, and the extent to which differently-timed responses to a stimulus can be measured.

Another important consideration is the fact that responses in auditory regions are not only influenced by the amplitude of the scanner noise, but by other parameters, such as its perceived continuity: auditory cortex responds strongly to pulsed noises in the frequency ranges associated with typical gradient switching, and thus typical EPI sequences make for particularly effective stimulation (Giraud et al., 2000; Harms and Melcher, 2002; Seifritz et al., 2003; Tanaka et al., 2000). Thus, a second approach for addressing some of the issues faced in auditory fMRI studies is to change the qualitative nature of the acoustic noise. This approach was taken by Seifritz et al. (2006), who developed an EPI sequence that emits continuous noise (rather than pulsed) by implementing a quasi-continuous gradient switching pattern. The authors presented audio recordings of the noise generated by both types of sequences to participants and recorded neural responses using a sparse imaging paradigm. They found that conventional EPI produced stronger responses than the continuous noise EPI. Additionally, responses to pure tones in auditory cortex were greater when measured with continuous noise EPI relative to conventional EPI. These results emphasize that the nature or quality of acoustic stimulation from the scanner, and not just its average loudness, must be considered in auditory fMRI studies.

Although changing the acoustic characteristics of the scanner noise effectively boosts BOLD responses in auditory areas for some stimuli, it still leaves open the possibility of interference by energetic masking, and may also lead to extra challenges of listening effort, especially for more complex (e.g., linguistic) stimuli. One way to mitigate these effects is to use active noise control to minimize the effects of scanner noise (Chambers et al., 2007; Hall et al., 2009). Here we adopt an alternate approach to reducing the impact of acoustic noise by using an EPI sequence that is sufficiently quiet to allow participants to easily perceive auditory stimuli, even in the presence of pulsed scanner noise (Schmitter et al., 2008). In this sequence, acoustic noise is minimized by using a constant phase encoding gradient and a sinusoidal readout echo train to produce a narrow-band acoustic frequency spectrum. The scanner-specific frequency response function can be measured using an MR-compatible microphone placed inside the magnet bore. It is then possible to choose a readout gradient switching frequency (within the limits imposed by BOLD fMRI) that results in a lower acoustic response based on this frequency response function. In addition, the clicking noise of the slice-selection gradient is reduced by choosing a lower slew rate.

This modified gradient switching scheme can reduce the acoustic noise of EPI by up to 20–30 dB compared to trapezoidal EPI using the same imaging parameters. However, it may also influence data quality. For example, the longer EPI readout duration required by using a slower gradient switching frequency would be expected to exacerbate susceptibility effects near tissue boundaries, such as in inferior temporal and orbital frontal regions (Devlin et al., 2000; Ojemann et al., 1997). In addition, the nonuniform sampling of *k*-space requires an adaptation of standard image reconstruction software, and because of the sinusoidal readout gradient, the resulting images are also smoother than those from a standard sequence.

The primary aim of the current study is to evaluate the data provided by this new sequence relative to existing EPI sequences. We chose to do so using auditory stimuli that result in robust and replicable patterns of activation in well-known regions of cortex based on several previous studies (Davis et al., 2007; Mummery et al., 1999; Rodd et al., 2005).

## Method

### Participants

Six healthy right-handed adults aged 20–26 years (3 females) participated in this study. All were native English speakers with self-reported normal hearing and no history of neurological problems. Written consent was obtained from all participants on a protocol approved by the local ethics committee.

### Materials

The materials consisted of a set of unambiguous sentences created for Rodd et al. (2005). Sentences ranged in duration from 1.14 to 3.58 s and contained simple declarative statements (e.g., “The police returned to the museum.”). The 120 sentences used were divided into 4 groups of 30, matched for duration, naturalness, imageability, and number of words using Match software (Van Casteren and Davis 2007), available from http://www.mrc-cbu.cam.ac.uk/people/maarten.van-casteren/mixandmatch.html. For each sentence, a probe word was generated for use in a behavioral task. Half of these probe words were semantically related to the sentence, and half were unrelated.

For a baseline condition, sentences matched in duration to the experimental sentences were used to create signal-correlated noise (SCN) (Schroeder, 1968). These stimuli have the same overall amplitude envelope and spectral profile as the original sentences but are lacking spectral detail, and are therefore entirely unintelligible. They thus provide a good control for the acoustic stimulation and temporal pattern of the sentences without conveying any linguistic information.

### Procedure

Participants were instructed to attend to each stimulus, and after each sentence respond to the probe word on the screen. In the case of the real sentences, the probe word had a 50% probability of being semantically related to the just-heard sentence; participants indicated whether or not the probe word was semantically related to the sentence using a button-press response. For example, for the sentence “There were beer and cider on the kitchen shelf,” a related probe word might have been “drink”. For the SCN trials, the word “left” or “right” appeared on the screen, and participants were instructed to press the appropriate button. Participants were situated in the magnet and familiarized with this procedure through a short practice session, during which we also ensured the sentences were being presented at a comfortable listening level. Participants were informed that they would be performing the same task four times, but that due to different imaging parameters the scanner noise would differ. In all cases they were instructed to ignore the scanner noise as much as possible and concentrate on listening to the auditory stimuli.

### Image acquisition

All images were acquired on a Siemens 3 T Tim Trio scanner (Siemens Medical Systems, Erlangen, Germany). The four EPI sequences used to collect data on the experimental paradigm are listed in Table 1 and described below. In addition to EPI data, we cquired a T1-weighted structural image for each participant using an MPRAGE sequence (TR = 2250 ms, TE = 2.99 ms, TI = 900 ms, flip angle = 9°, FOV = 256 mm × 240 mm × 160 mm, voxel size = 1 mm × 1 mm × 1 mm). In addition, field map data (2D structural and phase difference images) were acquired using a standard double echo GE sequence (TE1/TE2 = 5.19/7.65 ms; TR = 400 ms; flip angle = 60°, slice thickness = 3 mm; matrix size = 64 × 64; in-plane resolution = 3 × 3 mm; total acquisition time = 54 s). The phase difference images were unwrapped and converted into magnetic field maps (Jenkinson, 2003).

#### Standard EPI sequence

We acquired 32 slices in axial oblique orientation (i.e., angled to ensure the eyeballs were not on the same plane as auditory cortex to avoid possible Nyquist ghost artifacts), collected in a descending order, with a .75 mm gap between slices (TR = 2.8 s, TA = 2.8 s, TE = 30 ms, flip angle = 90°, FOV = 192 mm × 192 mm, matrix = 64 × 64 mm, voxel size = 3 × 3 × 3 mm, bandwidth = 2230 Hz/Px). We ensured full temporal lobe coverage in all participants, but did not cover portions of superior parietal lobe in participants with larger heads. Unless noted below these parameters were consistent across all sequences tested.

#### Sparse EPI sequence

The Sparse sequence used a sparse or clustered acquisition sequence commonly employed in fMRI studies of auditory processing. This approach relies on the fact that, due to the lag inherent in the hemodynamic response, the peak BOLD response to a stimulus occurs several seconds after the stimulus, and can thus be captured by a scan occurring later in time. The imaging parameters were identical to the standard sequence, with the exception that we used a TR of 11.2 s. Given the TA of 2.8 s, this left 8.4 s of silence in which we presented a single sentence or SCN, allowing the item to be presented in the absence of echoplanar scanner noise. The presentation of the sentences and SCN was timed so that the midpoint of each stimulus occurred 5 s before the midpoint of data acquisition in order to maximize detection of the peak hemodynamic response.

#### Quiet EPI sequence

This sequence is described in more detail in Schmitter et al. (2008). The principle behind this sequence is that because the acoustic response of gradient coils changes as a function of the gradient coil switching frequency, changing the gradient switching frequency to one with a low acoustic response can significantly reduce acoustic noise. The quiet sequence therefore involved gradient switching g(*t*) which is characterized by a narrow-band input spectrum G(*f*), located in a frequency interval where the frequency response function showed a local minimum, judged using the individual frequency response profile measured for the empty scanner using an MR-safe optical microphone (Sennheiser MO 2000, Sennheiser GmbH & Co. KG, Wedemark, Germany). Based on these measurements a readout bandwidth of 1220 Hz/Px was chosen, along with a TE of 44 ms. Finally, the quiet sequence also used sinusoidally switched readout gradients, with the phase encoding gradient switched constant. This combination results in an S-shaped trajectory through *k*-space, which was regridded using a custom software implementation. The sinusoidal readout gradient leads to smoother image reconstruction in the readout direction. Note that because the regridding procedure is identical for all image volumes, it does not result in additional signal variance over time. Sound levels measured inside the scanner indicated a considerable sound level reduction of approximately 20 dBA compared to the Standard sequence. Finally, as noted previously, the subjective quality of the scanner noise can influence the measured activity in auditory cortex. In our study, the perceived continuity of the Quiet sequence was similar to that of the Standard and Matched Standard (see below) sequences; that is, the reduction in overall acoustic amplitude would be expected to reduce the energetic masking, but responses that relate to the nature of the background noise (Seifritz et al., 2006) are unlikely to be significantly affected. This is an advantage in that, having equated the pulsatile nature of the scanning sequence across sequences, we can more confidently attribute changes in activity to the differences in acoustic noise levels. At the same time, there is a possibility that an EPI sequence that was not only acoustically quiet but also continuous would afford even greater sensitivity.

#### Matched Standard EPI sequence

There are two significant differences between the Standard and Quiet sequences: (1) the Quiet sequence has a longer TE, and a narrower bandwidth (1220 Hz/Px as opposed to 2230 Hz/Px); and (2) the Quiet sequence uses a different type of readout gradient, requiring custom reconstruction. Thus, if differences were found between the Quiet and Standard sequences, it would be difficult to know to which parameter we could attribute the difference. To address this issue we also collected data using the Matched Standard sequence, which is identical to the Standard sequence, with the exception that the duration of the EPI readout was matched to the Quiet sequence by choosing the same bandwidth of 1220 Hz/Px; thus, the frequency of the readout gradient was identical to that of the Quiet sequence.

### Signal-to-noise ratio measurements

To estimate the temporal signal-to-noise ratio (SNR) for each sequence, 6 white matter voxels (MNI coordinates: [−27 −18 32], [−22 39 4], and [−20 18 26] in the left hemisphere, along with their right hemisphere counterparts) were selected from which to sample the signal. Each voxel had at least a 97% probability of being white matter according to the tissue probability maps distributed with SPM, and their location in white matter was verified on the structural images for individual participants in the current study. For each sequence, the data from each voxel were extracted from the unsmoothed normalized images, and baseline corrected using a 5th order cosine basis set to remove low-frequency drifts. The SNR was then calculated by dividing the mean of the timeseries by its standard deviation (Krüger and Glover, 2001). For each sequence the SNR was calculated for each of the 6 voxels for each participant, and then the SNR values were averaged to provide a single SNR value for each sequence for each participant.

### BOLD sensitivity maps

BOLD sensitivity maps were calculated from the field maps and the specific acquisition parameters of the Standard and Quiet sequences using the theoretical framework described by De Panfilis and Schwarzbauer (2005) with the extension proposed by Weiskopf et al. (2007) to account for the effect of susceptibility gradients in readout direction. Because the Standard and Sparse sequences differ only in TR, the BOLD sensitivity maps are identical. In addition, the estimated sensitivity maps for the Quiet and Matched Standard sequences can be considered identical, as the relevant imaging parameters were identical and the effect of the nonuniform *k*-space sampling used in the Quiet sequence is negligible.

### fMRI analysis

Image preprocessing and statistical analyses were performed using SPM5 (Wellcome Trust Centre for Neuroimaging, London, UK). The first 9 images for the three continuous sequences and the first 3 images for the sparse sequence were discarded to allow for T2 saturation effects. Low-frequency drifts were removed with highpass filtering (with a cutoff period of 90 s) and autocorrelations were modeled using a first-order autoregressive model. Images for each participant were realigned to the first image in the series (Friston et al., 1995), corrected for effects of magnetic field inhomogeneity (Cusack et al., 2003) and coregistered with the structural image (Ashburner and Friston, 1997). The transformation required to bring a participant's images into standard MNI152 space was calculated using MNI-space tissue probability maps (2 × 2 × 2 mm) included with SPM5 (Ashburner and Friston, 2005), and these warping parameters were then applied to all functional images for that participant. During normalization the data were interpolated to 2 mm isotropic voxels using trilinear interpolation. Prior to statistical analysis the data were spatially smoothed with a 10 mm FWHM isotropic Gaussian kernel.

Data were initially analyzed separately for each participant, using a separate general linear model for each scanning sequence. Each event of interest (sentence or SCN) was convolved with a standard hemodynamic response function to create the repressors used in the model. This was specified as an event with no duration occurring at the middle of each stimulus. The 6 motion parameters obtained during realignment were included in the model as additional regressors. Following analysis on the subject level, images containing the contrasts of parameter estimates for each subject were entered into second-level group analyses. To ensure fair comparison for results across sequences despite differences in sensitivity and signal dropout, we used the same explicit brain mask (distributed with SPM) for all analyses. We did not use a proportional threshold for the fMRI data but analyzed all voxels included in the mask.

For comparisons among the four sequences we examined both parameter estimates and *t*-statistics derived from the first-level (single-subject) analyses. We examined *t*-statistics in addition to parameter estimates because they give an indication as to the effect size relative to the variability present (which we expected might differ by sequence). For example, due to the lack of spin history effects, images acquired with a sparse sequence tend to exhibit higher overall levels of signal, but also more variability due to the reduced number of observations.

Images are displayed on an MNI-space template brain included with MRIcron (Rorden and Brett, 2000), available from http://www.cabiatl.com/mricro/mricron/. Coordinates listed for maxima are in MNI152 space.

## Results

### Behavioral performance

We first examined accuracy on the behavioral task in all four scanning sequences, which was ≥ 93% correct in all cases. Accuracy did not differ as a function of scanning sequence, evidenced by a Friedman nonparametric test [*χ*^2^(3) = 2.32, *n.s.*], indicating that participants were able to accurately perceive the speech stimuli present during each scanning sequence. However, we note that equal intelligibility can still be achieved through different amounts of listening effort, which we expect to be the case when auditory stimuli are presented in the context of background noise.

### Signal-to-noise ratio

The sinusoidal readout gradient used in the Quiet sequence leads to an increase of the effective voxel size in readout direction, which translates into a linear increase in the SNR. Therefore, we first estimated the expected change in SNR based on this difference, relative to the Matched Standard sequence, which in all other ways is identical. The relative SNR gain, compared to the trapezoidal gradient switching schemes used in the other sequences, can be calculated from the areas under the respective gradient waveforms to give $rSNR=\frac{\pi }{2}\left(1-T_{\text{s}}/T_{\text{g}}\right)-1$, where *T*_s_ is the gradient ramp time and *T*_g_ the total duration of a trapezoidal gradient lobe. Compared to a *T*_s_/*T*_g_ = .15 (Matched Standard sequence), the expected SNR gain of the Quiet sequence is 34%.

**Fig. 1:**
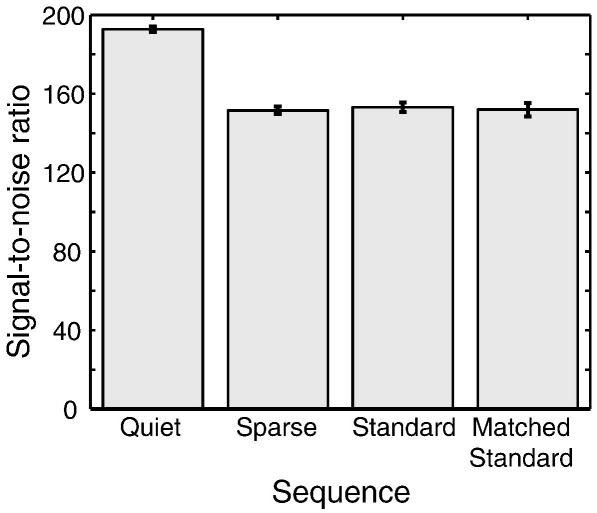
Signal-to-noise ratios calculated for the four EPI sequences tested. Error bars represent 1 standard error of the mean with between-subjects variance removed, suitable for within-subjects comparisons (Loftus and Masson, 1994). The increase in SNR for the Quiet sequence is due to its increased smoothness (see text for details); this effect is negligible in the activation analyses because of the smoothing applied during preprocessing.

Signal-to-noise ratio (SNR) values calculated based on extracted data for each sequence are plotted in Fig. 1. As expected, due to increased intrinsic smoothness, the Quiet sequence showed significantly higher SNR values. To assess whether the differences in values were significant, we submitted these data to a within-subjects one-way analysis of variance (ANOVA) to test for a main effect of sequence, which was significant, *F*(3,15) = 52.6, *p* < .001, MSE = 701.9. We then conducted post-hoc *t*-tests between the Quiet sequence and all other sequences; these confirmed that, the Quiet sequence had a higher SNR than all of the other sequences, all *t*s > 8.9 and all *p*s < .001. The relative change in SNR between the Matched Standard and Quiet sequences was 27%, which is in reasonably good agreement with the theoretical gain described above. Although the increase in SNR was expected due to the increased smoothness inherent in the Quiet sequence, it should be noted that this SNR difference in the unsmoothed data becomes negligible in the smoothed data used in the fMRI analyses, as the increase in the effective voxel size is small compared to the dimension of the Gaussian smoothing kernel (10 mm FWHM).

### BOLD sensitivity

**Fig. 2:**
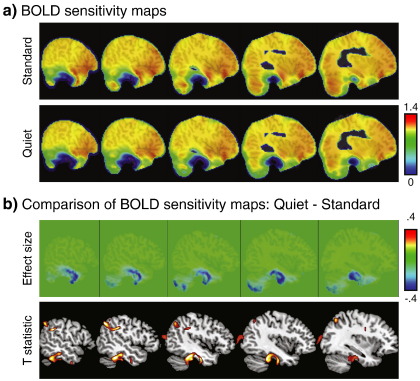
(a) Mean BOLD sensitivity maps for the Standard and Quiet sequences (which are identical for the Sparse and Matched Standard sequences, respectively). (b) Comparison of BOLD sensitivity maps. The effect size from the subtraction is shown at the top: warmer colors indicate voxels where the Quiet sequence shows increased sensitivity, and cool colors show decreased sensitivity relative to the Standard sequence. The bottom shows the results of a paired-samples *t*-test on smoothed BOLD sensitivity maps (Standard > Quiet, voxelwise threshold of *p* < .0001 uncorrected, cluster extent of 50 voxels, all reaching full brain significance for cluster extent).

We calculated BOLD sensitivity maps for each subject based on spatially normalized field maps, and then created group average maps, shown in Fig. 2a. This was done for the Standard and Quiet sequence (as noted previously, these are identical to those for the Sparse and Matched Standard, respectively). Although both sequences exhibit lower signal around the temporal lobes, this was most pronounced in the Quiet sequence. Shown in Fig. 2b is the comparison of the BOLD sensitivity maps across sequence. The absolute difference in sensitivity is shown in the top panel; there were no regions that showed more sensitivity for the quiet sequence, but several regions that showed less sensitivity (cool colors). To quantify this difference we performed a pairwise *t*-test to compare the two sets of BOLD sensitivity maps across participants. The maps were smoothed with a 10 mm FWHM isotropic Gaussian kernel prior to analysis to match the fMRI data. The results in the bottom portion of Fig. 2b, and listed in Table 2, show significant differences in sensitivity using a voxelwise threshold of *p* < .0001 (uncorrected) and a minimum cluster extent of 50 voxels which in all cases led to clusters which were whole-brain FWE-corrected for significance using cluster extent, *p* < .05 [FWHM = 14.7 mm × 12.9 mm × 11.5 mm, resel count = 1216.4]. Differences in BOLD sensitivity were widespread, and included inferior temporal and orbital frontal regions where the sensitivity was lowest. Whole-brain maps of BOLD sensitivity and differences are shown in Fig. S1–S4.

### Acoustic activity

**Fig. 3:**
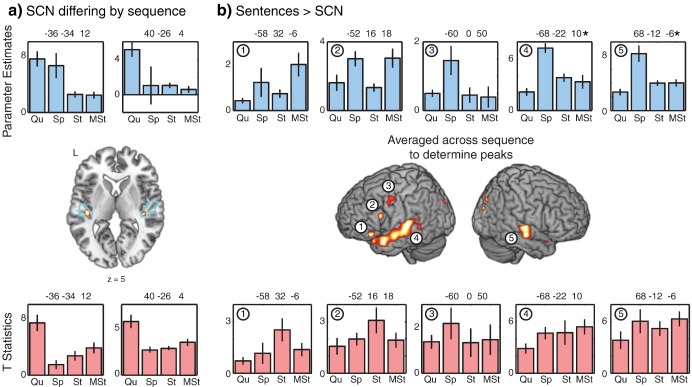
(a) Within cytoarchitectonically-defined bilateral primary auditory cortex (blue outline), we conducted a second-level within-subjects *F* test to identify voxels in which activity for SCN (relative to the mean over scans) differed significantly by imaging sequence. For two peaks resulting from this contrast, parameter estimates (top) and *t*-statistics (bottom) were extracted to illustrate the data driving the effect. (b) Whole-brain analysis to look for regions showing more activity for Sentences than SCN, averaged over scanning sequence, *p* < .005 voxelwise threshold corrected for whole-brain significance using cluster extent (*p* < .05). For five peaks resulting from this contrast, parameter estimates and *t*-statistics were extracted to examine the effects of imaging sequence on neural activity; *indicates significant ANOVA for a difference by sequence. MNI coordinates for extracted maxima are given above each plot. Abbreviations: Qu = Quiet, Sp = Sparse, St = Standard, MSt = Matched Standard sequence. Error bars represent 1 standard error of the mean with between-subjects variance removed, suitable for within-subjects comparisons.

We used the SPM anatomy toolbox (Eickhoff et al., 2005) to create a binary mask encompassing regions TE1.0 and TE1.1 of bilateral primary auditory cortex (Morosan et al., 2001), outlined in blue in Fig. 3a. Regions included in the mask had at least an 80% chance of belonging to these subdivisions based on cytoarchitectonic characteristics. We then conducted a within-subjects ANOVA to identify voxels in which the main effect of SCN relative to the mean over scans differed by sequence type, shown in Fig. 3a. We used a cluster-defining voxelwise threshold of *p* < .005 (uncorrected), and FWE-corrected for set-level significance within the search volume using cluster extent *p* < .05 (Worsley et al., 1992). For the peak voxel in each cluster, we then extracted the parameter estimates for each of the four sequences, shown in the top of Fig. 3a, to illustrate the nature of the difference between sequences. We also extracted the *t*-statistics from the first-level single-subject analysis, plotted along the bottom. These results indicate that in primary auditory regions the Quiet sequence gave the most robust response to SCN stimuli.

### Speech activity

A second comparison of interest was the increase in activity when participants heard sentences compared to when they heard SCN, a contrast which results in robust language-related activation when data are collected using a sparse scanning sequence (Davis et al., 2007; Rodd et al., 2005; Schwarzbauer et al., 2006). Results for the Sentences > SCN contrast averaged across the four scanning sequences are shown in Fig. 3b and listed in Table 3, using a cluster-defining voxelwise threshold of *p* < .005 (uncorrected), and whole-brain FWE-corrected for significance using cluster extent, *p* < .05 [FWHM = 9.4 mm × 9.7 mm × 9.6 mm, resel count = 2653.5].

To examine whether the Sentences > SCN contrast differed as a function of scanning sequence we extracted parameter estimates and first-level *t*-statistics for each participant from five of the peak voxels identified by the Sentences > SCN contrast, and subjected each to a one-way within-subjects ANOVA, Bonferroni-corrected to control for multiple comparisons within each dependent measure (i.e. separately for parameter estimates and *t*-statistics). For the parameter estimates, there were significant effects of sequence in the left posterior superior temporal gyrus (STG) [−68 −22 10: *F*(3,15) = 14.4, *p* < .005] and right posterior STG [68 −12 −6: *F*(3,15) = 16.2, *p* < .005]. None of the regions showed a significant effect of sequence on first-level *t*-statistics (all other *p*s > .05).

**Fig. 4:**
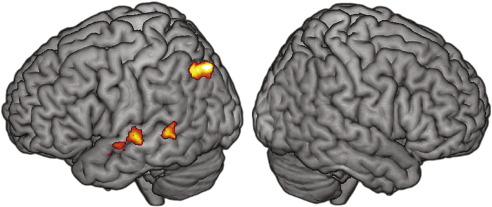
Effects of listening effort. Sentences > SCN activity which is significantly increased for the Standard sequence greater than the Quiet sequence, voxelwise *p* < .005, corrected for whole-brain significance (*p* ≤ .052) using cluster extent.

As noted previously, even sentences in which word report is equivalent can differ in the amount of effort required to achieve this level of intelligibility. To look for neural correlates of listening effort, we conducted a second-level paired-samples *t*-test comparing the Sentences > SCN parameter estimates for the Standard and Quiet sequences, under the assumption that the acoustically louder Standard sequence would be more effortful than the Quiet sequence, and thus result in increased neural activity. This comparison is shown in Fig. 4, with maxima listed in Table 4. We used a cluster-defining voxelwise threshold of *p* < .005 (uncorrected), and whole-brain FWE-corrected for significance using cluster extent, *p* ≤ .052 [FWHM = 8.9 mm × 9.0 mm × 8.9 mm, resel count = 3305.9]. This analysis revealed several significant peaks along left superior temporal cortex, as well as left inferior parietal cortex (extending into angular gyrus).

**Fig. 5:**
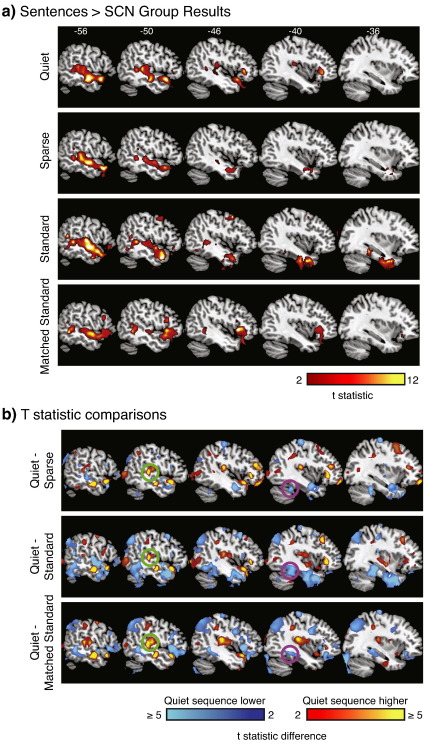
Qualitative comparison of results for the four imaging sequences. (a) Whole-brain group analyses for Sentences > SCN for each of the four imaging sequences, voxelwise *p* < .005, corrected for whole-brain significance (*p* < .05) using cluster extent. (b) Differences in group *t*-statistics between the Quiet sequence and the other three sequences. All voxels shown differ by 2 or more. Cool colors indicate voxels in which the Quiet sequence showed lower *t*-statistics than other sequences; warm colors where it showed higher *t*-statistics than other sequences. Highlighted are primary auditory cortex (green circles) and inferior temporal/fusiform gyrus (magenta circles).

Finally, to assess the qualitative effect of different sequences on the Sentences > SCN contrast at a group level, we performed group (random effects) analyses for each of the four sequences for this contrast. For each sequence the significant effect of Sentences > SCN is shown in Fig. 5a, with a cluster-defining voxelwise threshold of *p* < .005 (uncorrected), and whole-brain FWE-corrected for significance using cluster extent, *p* < .05. Fig. 5b shows the arbitrarily-thresholded subtraction of group *t*-statistics, demonstrating differences in group *t*-statistics for the Quiet sequence relative to the other three sequences. Of particular interest are the higher *t*-statistics near primary auditory areas (green circles) and lower *t*-statistics in inferior temporal regions (magenta circles) in the Quiet sequence relative to all other sequences. These qualitative observations are consistent with our quantitative comparisons, and support increased sensitivity of the Quiet sequence near primary auditory areas, and reduced sensitivity in inferior temporal regions.

## Discussion

There are multiple challenges associated with using conventional echoplanar fMRI sequences to study neural responses to auditory stimuli due to acoustic scanner noise. Although for many years sparse imaging has proved to be a valuable approach, information regarding the timecourse of the BOLD response is lacking in these studies. An ideal sequence would be sufficiently quiet so as not to challenge auditory processing, and yet provide continuous information regarding brain activity. In the current report we set out to see whether the quiet EPI sequence introduced by Schmitter et al. (2008) might fulfill this role. Previously it has been shown that this sequence increases sensitivity in primary auditory areas in response to pure tone stimulation (Schmitter et al., 2008); in the current report we extended these results by including a greater number of comparison sequences, and by assessing the sensitivity of the Quiet sequence to both nonlinguistic auditory stimuli (i.e., SCN) and sentences. These are discussed in turn below.

### Responses to SCN in primary auditory cortex

The neural response of auditory cortex to acoustic scanner noise has been a subject of interest for some time. It is clear that the considerable sound produced by echoplanar imaging produces robust responses in primary auditory cortex and surrounding areas (Bandettini et al., 1998; Hall et al., 2000; Talavage et al., 1999), and that this elevated response can reduce task-related activation (Elliott et al., 1999; Gaab et al., 2007; Haller et al., 2005). We hypothesized, therefore, that the Quiet sequence would show a greater response to basic task-related acoustic stimulation than the louder continuous sequences. This prediction was borne out: the Quiet sequence showed the largest response to SCN in cytoarchitectonically-defined primary auditory cortex.

Perhaps somewhat surprising is that the response was larger in these auditory regions in the Quiet sequence than the Sparse sequence, in which there is no scanner noise during stimulus presentation. One possible reason for this is that, for a given period of scanning time, the Quiet sequence collects more scans compared to the Sparse sequence. Even if all other factors were held constant, this substantial increase in degrees of freedom would result in a more reliable response measurement. Although these additional degrees of freedom are reflected in the single-subject *t*-statistics, they do not affect the parameter estimates, in which the Quiet sequence also appears to show a significant advantage. This may be due to variability in the timecourse of the BOLD response, either across participants (Aguirre et al., 1998) or across stimuli. That is, in a sparse imaging paradigm, the time of each stimulus relative to a scan is typically chosen so as to capture the BOLD response approximately 4–6 s after the event, and thus hopefully measure the peak response. However, because the timecourse of the response is not identical across stimuli or participants, it is possible for the single scan acquired in sparse imaging to miss the peak response. The continuous data acquisition afforded by the Quiet sequence may enhance the ability to detect the BOLD response from auditory regions. In principle, this may be especially true when longer stimuli, such as sentences, are used, as the estimation of when a discrete “event” occurs is not straightforward. However, if this was the case, we would expect the Quiet sequence to consistently outperform the Sparse sequence (i.e., in all conditions), as it should always be able to give a better estimation of the hemodynamic response. Because of the lack of differences in the Sentences > SCN comparison (discussed below), then, this seems to be an unlikely explanation.

A third possible reason why the Quiet sequence showed greater auditory activation than the Sparse sequence is that, although the Sparse sequence does not involve scanner noise concurrent with the stimuli, it is still relatively loud (∼ 90 dBA). This may induce other phasic changes in the response of auditory cortex which our TR of 11.2 s (which in the context of sparse imaging is fairly moderate) would not permit to completely return to baseline. This may be particularly relevant for sparse imaging as with this type of acoustic stimulation significant onset responses (i.e. edge detection) might be expected for each scan (Chait et al., 2007; Herdener et al., 2007).

### Responses to sentences in frontal, temporal, and parietal cortices

To identify a sentence processing network, we first looked for the effect of Sentences > SCN averaged over scanning sequence. This analysis identified regions in frontal and temporal cortex that are in good agreement with a number of previous studies using the same comparison (Davis et al., 2007; Mummery et al., 1999; Rodd et al., 2005; Schwarzbauer et al., 2006). Within these regions we selected 5 maxima at which to examine the difference in responses between sequences. Only the left and right posterior STG regions showed a significant difference in parameter estimates among sequences, with the Sparse sequence providing the largest parameter estimates. All other regions showed statistically equivalent parameter estimates across sequence, which was also the case for all regions with the first-level *t*-statistics. These results suggest that, in contrast to what was observed in primary auditory areas, the response in these sentence processing regions is largely insensitive to the scanning sequence used. The higher parameter estimate for the Sparse sequence may be explained by the decay of spin history effects, associated with the pause between scans, which typically provide larger yet more variable signal. This conclusion is supported by the lack of a clear advantage for the Sparse sequence in the first-level *t*-statistics (in which scan-by-scan variance is taken into account).

One core sentence processing area not identified by our whole-brain analysis of Sentences > SCN is the left posterior inferior temporal gyrus and/or nearby fusiform gyrus (hereafter referred to together as inferotemporal cortex), a region that has been gaining in prominence in theories of speech processing (Hickok and Poeppel, 2007). In a voxel-based lesion-symptom mapping study, Bates et al. (2003) showed that damage to this region in stroke patients is associated with reduced auditory sentence comprehension. In neuroimaging studies activity in inferotemporal regions has been reported for spoken words > pseudowords (Binder et al., 2000; Orfanidou et al., 2006; see also the meta-analysis in Davis and Gaskell (2009)) and during comprehension of connected speech (Awad et al., 2007; Crinion et al., 2003). Perhaps most relevant for the current discussion, robust left inferotemporal activity in response to sentences has been observed in previous studies using materials similar to the ones in the current study (Davis et al., 2007; Rodd et al., 2005). As reviewed by Price (2000), activity in left inferotemporal cortex is common in word processing studies of both written and spoken language, including silent reading (words > false font), auditory and visual word retrieval, reading Braille, and several types of naming. The common activity across different tasks and modalities suggests a critical role in language processing. Based primarily on studies of single word processing, Price (2000) concluded that left inferotemporal cortex played a role in semantic processing. This is consistent with more recent studies of sentence processing in which sentences containing increased semantic ambiguity result in increased inferotemporal recruitment (Davis et al., 2007; Rodd et al., 2005). Together the available evidence makes a compelling case for left posterior inferotemporal cortex playing a critical role in semantic processing, and we thus expect its ubiquitous involvement in determining the meaning of connected speech.

The absence of inferotemporal activation in response to sentences in our current analysis is best explained by the significant signal dropout in this region in the Quiet and Matched Standard sequences caused by the longer duration of the EPI readout used in these sequences. In fact, the comparison of group results show that the Quiet sequence detected less sentence-related activity in this region than any of the other three sequences. Thus, the Quiet sequence appears to do as well as other sequences within most of these speech-processing regions, but is not sensitive to activations in inferotemporal cortex.

The lack of posterior inferotemporal activation due to susceptibility effects in our study highlights the difficulty of obtaining adequate signal in this region, even with standard EPI sequences, which may contribute to the lack of consensus regarding speech-related responses in posterior inferior temporal cortex in fMRI studies. Even with standard TEs (i.e., 30 ms for a 3 T scanner), inadequate active shimming may lead to substantial signal loss, which may go unnoticed. We reiterate the recommendation of Poldrack et al. (2008) that fMRI researchers check internally, and also report, average BOLD sensitivity and group coverage maps (e.g., the mask used in analysis) to provide an indication of the likelihood of detecting activations throughout the brain, particularly in regions prone to susceptibility artifact. An indication of the coverage provided by a mask (implicit, explicit, or a combination) is especially important if a proportional threshold is used in analysis. Given the increasing realization of the critical role played by inferior temporal regions in speech processing, this step would be extremely beneficial in interpreting the sometimes inconsistent fMRI results from this region.

Finally, we have suggested above that even if stimuli can be perceived in the presence of scanner noise, the additional effort required to achieve this perception will result in increased cognitive demand. For example, Davis and Johnsrude (2003) used three different acoustic manipulations to degrade sentence stimuli, and found that increased activity in large portions of left temporal, prefrontal and premotor cortices was associated with listening to the more degraded sentences. In the current study we hypothesized that due to increased energetic masking, additional neural activity would be associated with successful sentences comprehension during the Standard sequence relative to the Quiet sequence. Consistent with this prediction, we found significantly more speech-related activity in several areas of left temporal cortex, as well as left inferior parietal cortex. Although this finding must be interpreted with some caution due to the difference in scanning sequence, these areas did not show large differences in BOLD sensitivity, and thus we think it likely that these increases can be attributed to the different acoustic environments of the Standard and Quiet sequences. This finding underscores the importance of listening effort in interpreting data from auditory fMRI studies, and in particular the effects of standard EPI noise on required effort. Interestingly, contrary to Davis and Johnsrude (2003), we did not see significant increases in frontal regions associated with listening effort. This might be due to the relatively small sample size in the current study, or may instead reflect different processes required when acoustic degradation is intrinsic to the speech signal and hence originates from the same spatial location as the noise (Davis and Johnsrude, 2003) or spatially separate (as in the current study).

## Conclusions

Relative to standard continuous EPI sequences or popular sparse imaging approaches, the Quiet scanning sequence we evaluated (Schmitter et al., 2008) provides increased sensitivity to acoustic response in primary auditory regions, and a comparable response to sentences within a fronto-temporal speech network. The most notable disadvantage of the Quiet sequence was an increase in signal dropout in inferior temporal cortex associated with its longer EPI readout duration, and the resulting lack of sensitivity to language-related activation in this region. As a future improvement, parallel imaging acquisition techniques could be used to reduce the duration of the EPI readout, which would considerably improve its sensitivity of the Quiet EPI in regions affected by macroscopic magnetic field inhomogeneities. Future work comparing the effect of sound level reductions (as in the current study) and changes in perceived continuity (Seifritz et al., 2006) or active noise cancelling (Hall et al., 2009) would also be of interest.

It is important to emphasize that various types of stimuli may interact with acoustic scanner noise differently; one example of this being the different effects we have reported for SCN and Sentences > SCN. Thus, care should be taken when generalizing our findings to novel stimuli. However, the current results suggest that technical developments in acoustically quiet echoplanar imaging have the potential to increase sensitivity to the BOLD response for a wide variety of auditory stimuli. These approaches may be most helpful when acoustic-related activity in and near primary auditory cortex is of primary interest. In addition, they may have applicability in non-auditory tasks in service of increasing participant comfort and removing potential task confounds, provided areas prone to susceptibility effects are not of interest.

## Figures and Tables

**Table 1:** Summary of EPI sequences tested.

Sequence	TR (s)	TA (s)	TE (ms)	Bandwidth (Hz/Px)	# of scans	dBA
Standard	2.8	2.8	30	2230	284	90.2
Sparse	11.2	2.8	30	2230	71	n/a
Matched Standard	2.8	2.8	44	1220	284	n/a
Quiet	2.8	2.8	44	1220	284	67.4

Note: Sound levels were measured for the Standard and Quiet sequences with an MR-compatible microphone placed inside an empty scanner. Measurements were not taken for the Matched Standard sequence but it was uniformly judged to be louder than the Quiet sequence, and softer than the Standard sequence. During the Sparse sequence the only noise came from ambient scanner noise (e.g., helium pumps).

**Table 2:** Maxima of regions showing decreased BOLD sensitivity for the Quiet sequence compared to the Standard sequence.

Region	# of voxels	Coordinates			*Z* score
*x*	*y*	*z*
Sinus	2297	2	16	−26	6.08
L orbital frontal cortex		−12	22	−24	5.15
L ventral frontal (eyeball)		−26	42	−26	5.12
Ventromedial frontal cortex		−6	24	−24	5.06
Orbital frontal cortex		−2	66	−4	5.06
R inferior temporal cortex	1332	68	−26	−34	5.57
R fusiform gyrus		36	−22	−26	5.44
R medial inferior medial temporal		18	−26	−32	5.29
Supplemental motor area	4766	4	−2	66	5.30
R supplemental motor area		12	10	74	4.98
L supplemental motor area		−10	−4	68	4.94
R supplemental motor area		10	−10	62	4.64
L supplemental motor area		−18	−10	64	4.42
Lateral ventricles		2	0	26	4.38
R orbital frontal cortex	163	22	50	−24	5.28
L posterior inferior temporal/fusiform	1114	−48	−40	−26	5.09
L anterior inferior temporal/fusiform		−46	−12	−30	4.90
L posterior inferior temporal/fusiform		−50	−30	−20	4.58
L supramarginal gyrus	711	−60	−46	30	5.02
L inferior parietal lobe/angular gyrus		−50	−62	44	5.01
L superior parietal lobe		−30	−64	50	4.69
R posterior temporal lobe	297	56	−58	20	4.84
R inferior parietal sulcus	87	24	−70	50	4.18
L medial inferior temporal lobe	120	−22	−26	−38	4.07
L medial inferior temporal lobe		−18	−18	−30	4.04
L occipital gyrus	263	−44	−86	16	4.03
Anterior cingulate	60	4	40	10	3.86

Note: When areas of significant difference occur outside the brain (i.e. near brain/air boundaries), these are described by the nearest cortical landmark.

**Table 3:** Maxima of regions for Sentences > SCN contrast (averaged across scanning sequence).

Region	# of voxels	Coordinates			*Z* score
*x*	*y*	*z*
L anterior temporal cortex	5051	−62	18	−20	5.17
L posterior superior temporal gyrus		−68	−22	10	4.94
L inferior frontal gyrus pars opercularis and triangularis		−52	16	18	4.74
L ventral inferior frontal gyrus		−58	32	−6	4.24
R middle occipital gyrus	2657	44	−84	28	4.26
Calcarine fissure		6	−96	16	4.26
R posterior superior temporal gyrus	1104	66	−14	6	4.11
R superior temporal gyrus		68	−12	−6	4.02
R superior temporal gyrus		56	−26	6	3.07
L precuneus	483	−6	−50	10	3.68
R precuneus		6	−50	10	3.52
R anterior superior temporal gyrus	197	60	26	−18	3.65
R anterior superior temporal gyrus		54	18	−18	2.98
L middle occipital gyrus	202	−30	−84	34	3.58
L precentral gyrus	467	−60	0	50	3.35
L precentral gyrus		−50	0	50	3.16
L basal ganglia/thalamus	158	−16	−16	−12	3.34
		−14	−22	−4	3.10
		−22	−6	−4	2.85

**Table 4:** Maxima of regions showing effects of listening effort (sentence-related neural activity greater in Standard than Quiet sequence).

Region	# of voxels	Coordinates			*Z* score
*x*	*y*	*z*
L inferior parietal cortex	339	−36	−74	44	3.97
L angular gyrus		−40	−66	44	3.72
L inferior parietal cortex		−48	−60	48	3.40
L posterior middle temporal gyrus	209	−56	−46	8	3.71
L posterior middle temporal gyrus		−66	−44	0	3.18
L anterior superior temporal sulcus	148	−68	−14	2	3.27
L anterior superior temporal sulcus		−68	2	−8	3.21
L anterior superior temporal sulcus		−60	4	−14	2.78

## Appendix A Supplementary data

Fig. S1 Mean BOLD sensitivity maps for the Standard sequence overlaid on a template brain. Axial slices are shown from −35 mm to 60 mm. Blue regions around orbital frontal and inferior temporal cortex indicate regions of low sensitivity.

Fig. S2 Mean BOLD sensitivity maps for the Quiet sequence overlaid on a template brain. Axial slices are shown from −35 mm to 60 mm. Blue regions around orbital frontal and inferior temporal cortex indicate regions of low sensitivity.

Fig. S3 Absolute difference in BOLD sensitivity for the Quiet compared to the Standard sequence overlaid on a template brain. Axial slices are shown from −35 mm to 60 mm. Cool colors indicate regions where the Quiet sequence is less sensitive than the Standard sequence.

Fig. S4 SPM{*t*} map showing voxels in which the BOLD sensitivity for the Quiet sequence was significantly lower than that of the Standard sequence, using a cluster-defining threshold of *p* < .0001 (uncorrected) and a cluster extent of 50 voxels; all of the resulting clusters are whole-brain corrected for family wise error based on cluster extent, *p* < .001. Axial slices of a template brain are shown from −35 mm to 60 mm.
