# Supplementary material for: Evaluating an acoustically quiet EPI sequence for use in fMRI studies of speech and auditory processing
Source: Neuroimage. 2010 Oct 1;52(4-3):1410–9. doi: 10.1016/j.neuroimage.2010.05.015 (PMC2946564; doi:10.1016/j.neuroimage.2010.05.015)

**Supplemental Figures for Peelle JE, Eason RJ, Schmitter S, Schwarzbauer C, Davis MH (2010) Evaluating an acoustically quiet EPI sequence for use in fMRI studies of speech and auditory processing. *NeuroImage*. doi:10.1016/j.neuroimage.2010.05.015**

**Fig. S1.** Mean BOLD sensitivity maps for the Standard sequence overlaid on a template brain. Axial slices are shown from -35 mm to 60 mm. Blue regions around orbital frontal and inferior temporal cortex indicate regions of low sensitivity.

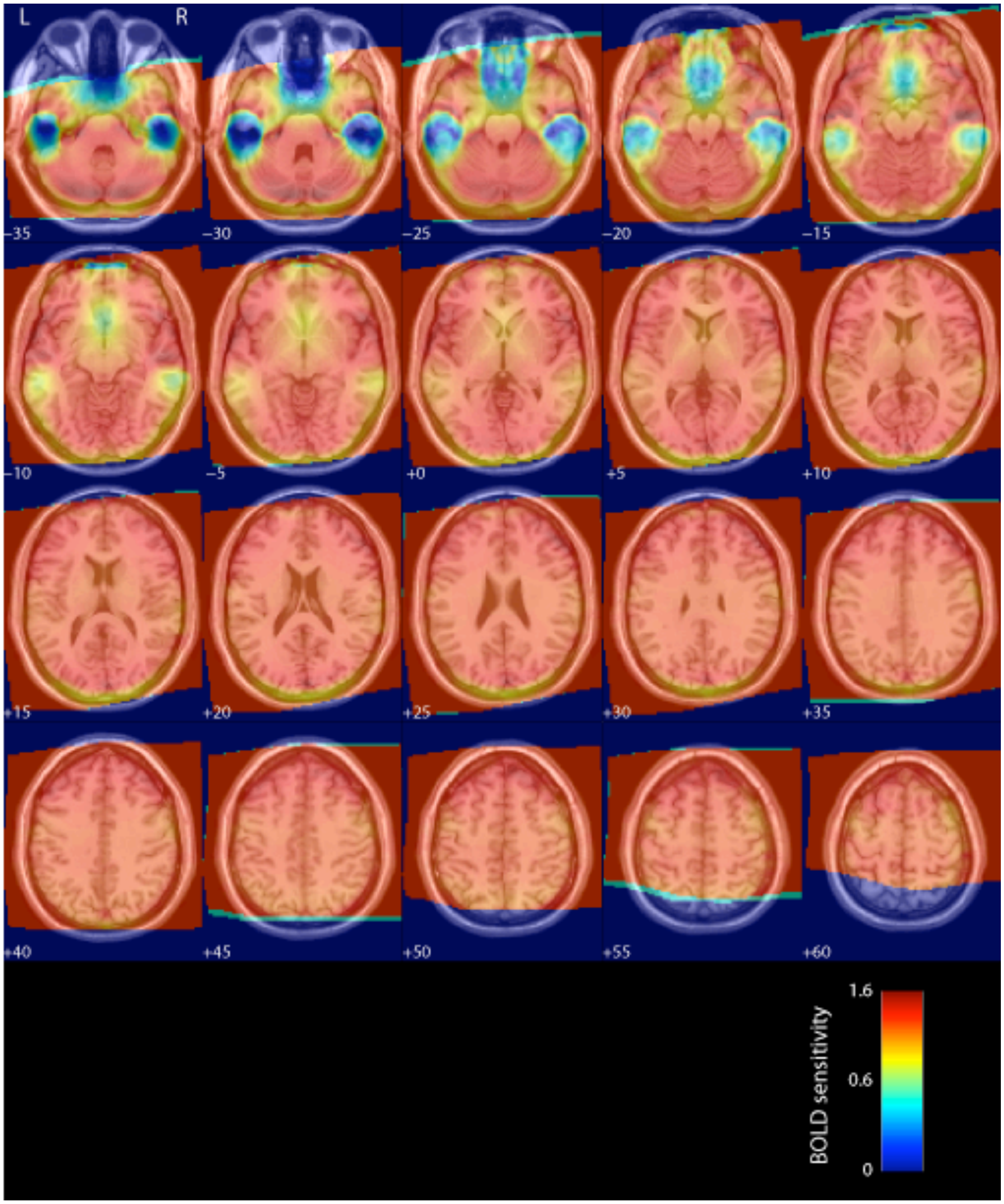

**Fig. S2.** Mean BOLD sensitivity maps for the Quiet sequence overlaid on a template brain. Axial slices are shown from -35 mm to 60 mm. Blue regions around orbital frontal and inferior temporal cortex indicate regions of low sensitivity.

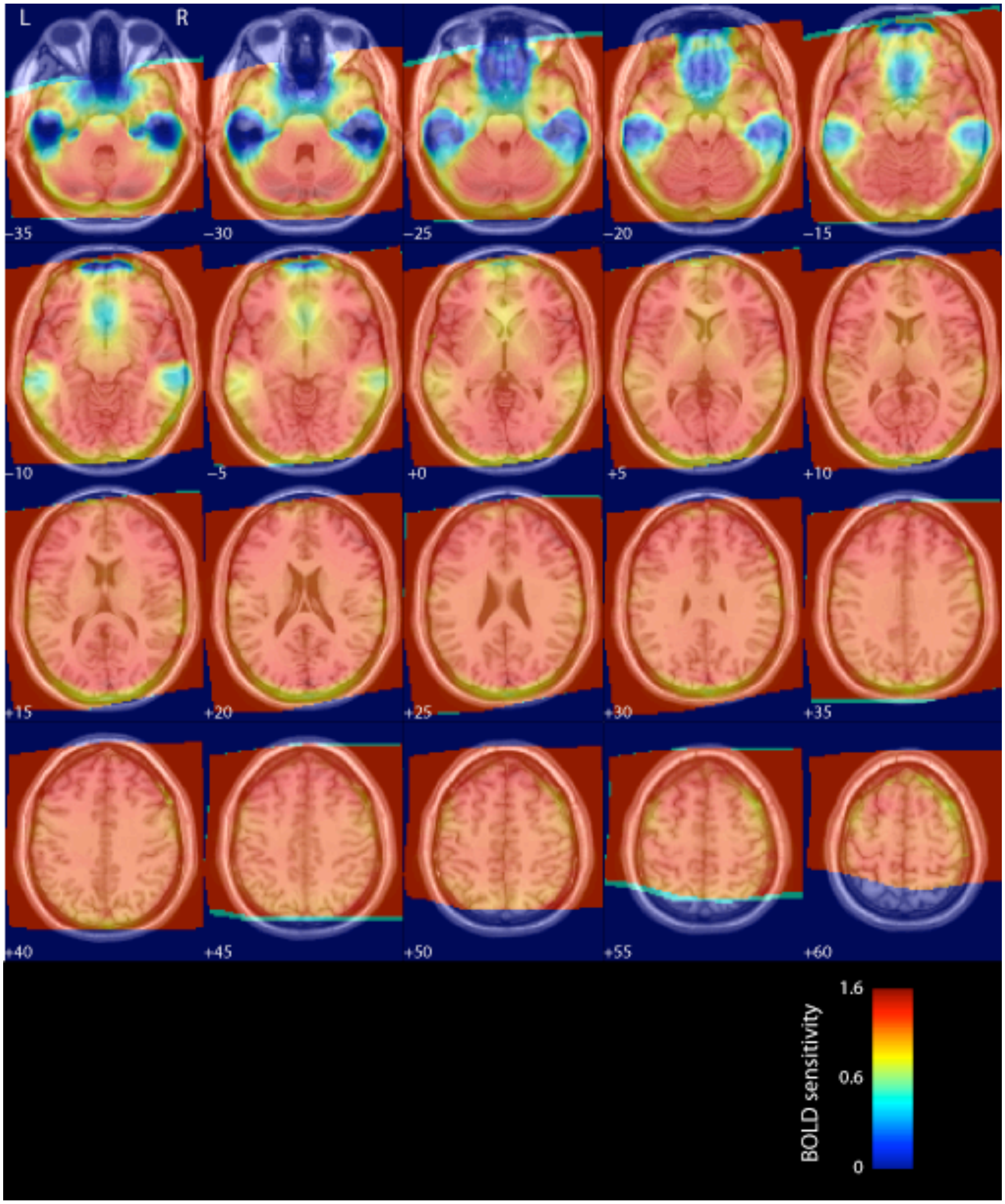

**Fig. S3.** Absolute difference in BOLD sensitivity for the Quiet compared to the Standard sequence overlaid on a template brain. Axial slices are shown from -35 mm to 60 mm. Cool colors indicate regions where the Quiet sequence is less sensitive than the Standard sequence.

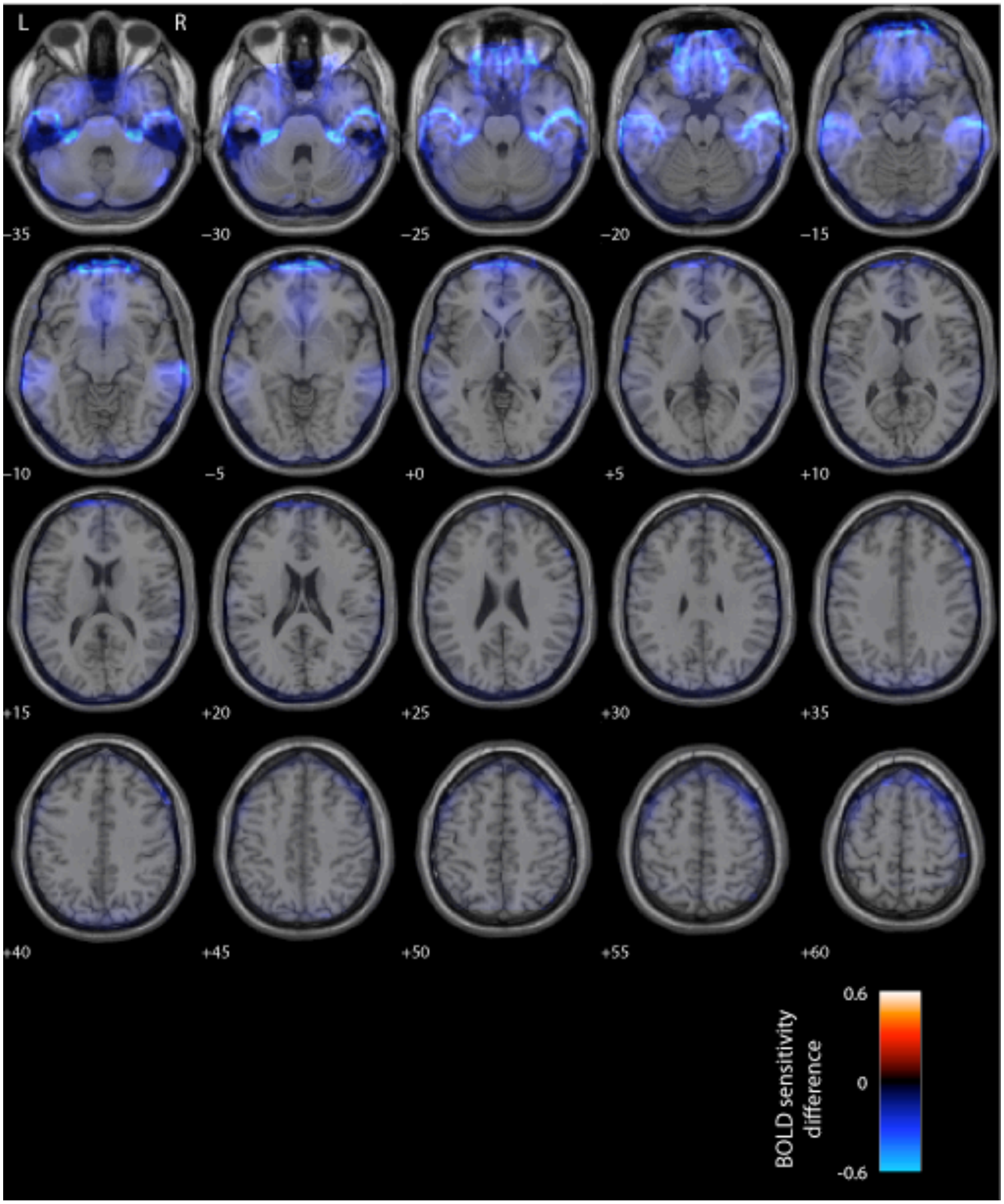

**Fig. S4.** SPM{t} map showing voxels in which the BOLD sensitivity for the Quiet sequence was significantly lower than that of the Standard sequence, using a cluster-defining threshold of $p < .0001$ (uncorrected) and a cluster extent of 50 voxels; all of the resulting clusters are whole-brain corrected for family wise error based on cluster extent, $p < .001$. Axial slices of a template brain are shown from -35 mm to 60 mm.

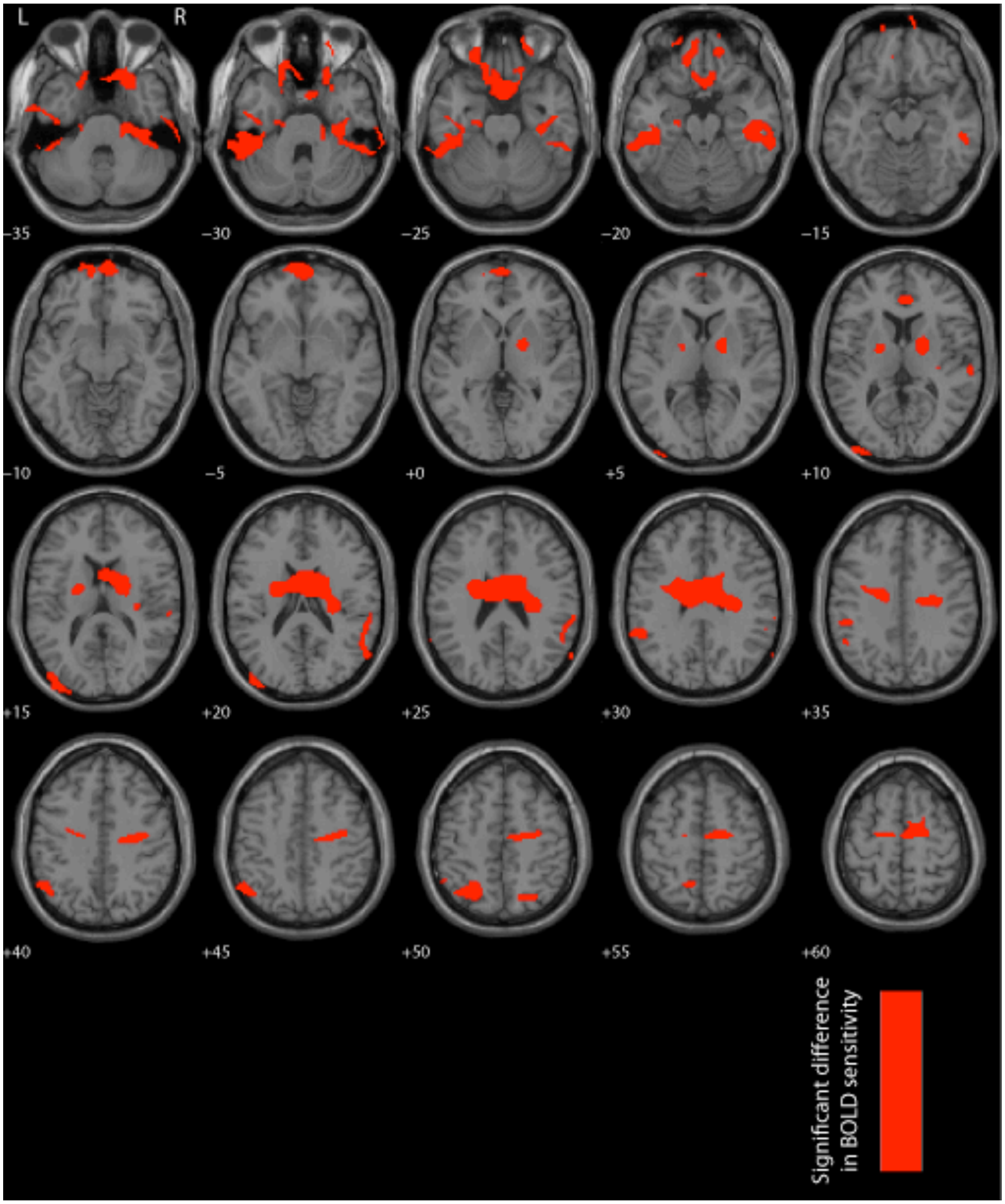

Supplement: Fig. S1 — Mean BOLD sensitivity maps for the Standard sequence overlaid on a template brain. Axial slices are shown from −35 mm to 60 mm. Blue regions around orbital frontal and inferior temporal cortex indicate regions of low sensitivity. [file mmc1.pdf]
